# An Automatic Needle Puncture Path-Planning Method for Thermal Ablation of Lung Tumors

**DOI:** 10.3390/diagnostics14020215

**Published:** 2024-01-19

**Authors:** Zhengshuai Wang, Weiwei Wu, Shuicai Wu, Zhuhuang Zhou, Honghai Zhang

**Affiliations:** 1 Department of Biomedical Engineering, Faculty of Environment and Life, Beijing University of Technology, Beijing 100021, China; 2 College of Biomedical Engineering, Capital Medical University, Beijing 100054, China; 3 Beijing Youan Hospital, Capital Medical University, Beijing 100054, China

**Keywords:** lung tumor, thermal ablation, improved cube mapping method, path planning, Pareto optimization

## Abstract

Computed tomography (CT)-guided thermal ablation is an emerging treatment method for lung tumors. Ablation needle path planning in preoperative diagnosis is of critical importance. In this work, we proposed an automatic needle path-planning method for thermal lung tumor ablation. First, based on the improved cube mapping algorithm, binary classification was performed on the surface of the bounding box of the patient’s CT image to obtain a feasible puncture area that satisfied all hard constraints. Then, for different clinical soft constraint conditions, corresponding grayscale constraint maps were generated, respectively, and the multi-objective optimization problem was solved by combining Pareto optimization and weighted product algorithms. Finally, several optimal puncture paths were planned within the feasible puncture area obtained for the clinicians to choose. The proposed method was evaluated with 18 tumors of varying sizes (482.79 mm^3^ to 9313.81 mm^3^) and the automatically planned paths were compared and evaluated with manually planned puncture paths by two clinicians. The results showed that over 82% of the paths (74 of 90) were considered reasonable, with clinician A finding the automated planning path superior in 7 of 18 cases, and clinician B in 9 cases. Additionally, the time efficiency of the algorithm (35 s) was much higher than that of manual planning. The proposed method is expected to aid clinicians in preoperative path planning for thermal ablation of lung tumors. By providing a valuable reference for the puncture path during preoperative diagnosis, it may reduce the clinicians’ workload and enhance the objectivity and rationality of the planning process, which in turn improves the effectiveness of treatment.

## 1. Introduction

Cancer remains a major threat to human health and is the main cause of death [1]. Lung cancer is the leading cause of death in the world among all types of cancer, posing a serious threat to human health [2]. The main treatment methods for lung cancer include pneumonectomy [3], radiation therapy [4], and chemotherapy [5]. In recent years, local thermal ablation therapy, with the advantages of minimal invasiveness, cost-effectiveness, and rapid postoperative recovery, has gradually developed into a new treatment method for patients with lung cancer who do not meet surgical conditions [6].

**Figure 1:**
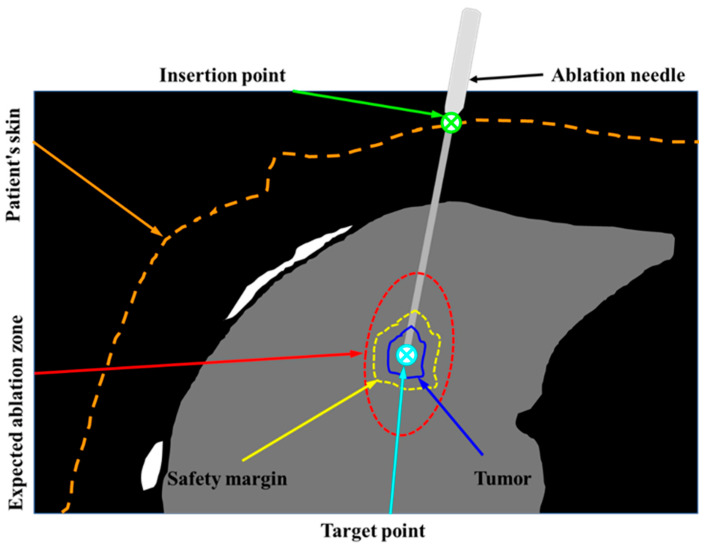
Schematic diagram of radiofrequency/microwave ablation of lung tumors. An ablation applicator (needle) is inserted percutaneously into the tumor to destroy the tumor cells in situ by heating. A heating-induced ablation zone is created which encompasses the tumor with a 5–10 mm safety margin.

Computed tomography (CT)-guided thermal ablation of lung tumors has been used in clinical settings. Under the guidance of CT images, a microwave (or radiofrequency) ablation needle is percutaneously inserted into a lung tumor as the heating source, and the lung tumor is heated to a high temperature within a few minutes to form complete coagulation necrosis, so as to destroy the tumor in situ (Figure 1). Local ablation of lung tumors can be divided into three stages: preoperative, intraoperative, and postoperative. Among them, preoperative puncture path (trajectory) planning in preoperative medical diagnosis is the key to successful ablation treatment, which can effectively avoid damage caused by improper ablation [7]. At present, clinical ablation needle puncture path planning is still highly dependent on the clinician’s personal experience. Clinicians manually mark the ablation needle puncture path based on layer-by-layer observation of two-dimensional (2D) CT sequences. A path-planning system may be used to interactively plan the ablation needle puncture path based on three-dimensional (3D) reconstruction of the patient’s thoracic cavity [8]. Ablation needle puncture path planning is a multi-objective (with multiple clinical constraints) optimization problem, and it is difficult to achieve the optimum by combining multiple clinical constraints based solely on the clinician’s experience [9]. Unreasonable planning of the puncture path increases the radiation dose borne by the patient (repeated needle insertions or calibrations), and may cause injury in severe cases [10]. Therefore, researchers have been working on the development of computer-aided preoperative path-planning techniques. This technology primarily accomplishes two objectives. Firstly, it mitigates the cognitive load and the extensive time investment required for clinicians in preoperative planning. Secondly, it underpins the enhancement of path rationality through objective, data-driven algorithms, thereby providing a reference for clinicians and improving the therapeutic effect of ablation.

For needle path planning, several methods have been proposed in the past few years. März et al. [11] proposed a semi-automated path-planning method that requires manual interaction by the clinician. Similarly, Stoll et al. [12] introduced an approach that combines manual with automated planning, taking into account distances from important organs during path planning. In order to achieve fully automated path planning, Villard et al. [13,14] and Baegert et al. [15,16,17] performed surface rendering on the patient’s organs and tissues, and regarded the line between the vertices of each polygon used for reconstructing the skin and the center of the tumor model as the potential puncture paths, and with the use of the Nelder–Mead simplex optimization algorithm and the constructed evaluation function, the optimal puncture paths were obtained. The accuracy and operational efficiency of this path-planning algorithm are heavily dependent on the resolution and reconstruction quality of the polygons in the surface rendering process, which limits the potential of the algorithm to some extent. The group of Schumann et al. [18,19,20] proposed an ablation path-planning algorithm to avoid the above shortcomings. The core of the algorithm is to take the centroid of the tumor as the origin, project cylindrically onto the patient’s skin surface, and then discretely sample the patient’s skin surface through the two-directional deflection angle in the process of projection so that a number of potential puncture points can be obtained on the patient’s body surface. The center of projection (tumor centroid) of this algorithm is usually not on the body’s central axis, making the discrete sampling of the deflection angle unevenly spaced across the patient’s skin surface, and therefore the accuracy of the potential puncture paths can be compromised. However, most of these methods are proposed for brain and abdominal tissues [21], and few are designed for thermal lung tumor ablation needle path planning. In this work, we proposed a method for automatic planning of lung tumor ablation needle paths, which can accurately and quickly provide clinicians with a personalized and reasonable puncture path reference. Our method provides reference value for preoperative diagnosis and treatment plans for lung tumor ablation, and provides a reliable reference procedure for the clinical puncture path.

## 2. Methods

**Figure 2:**
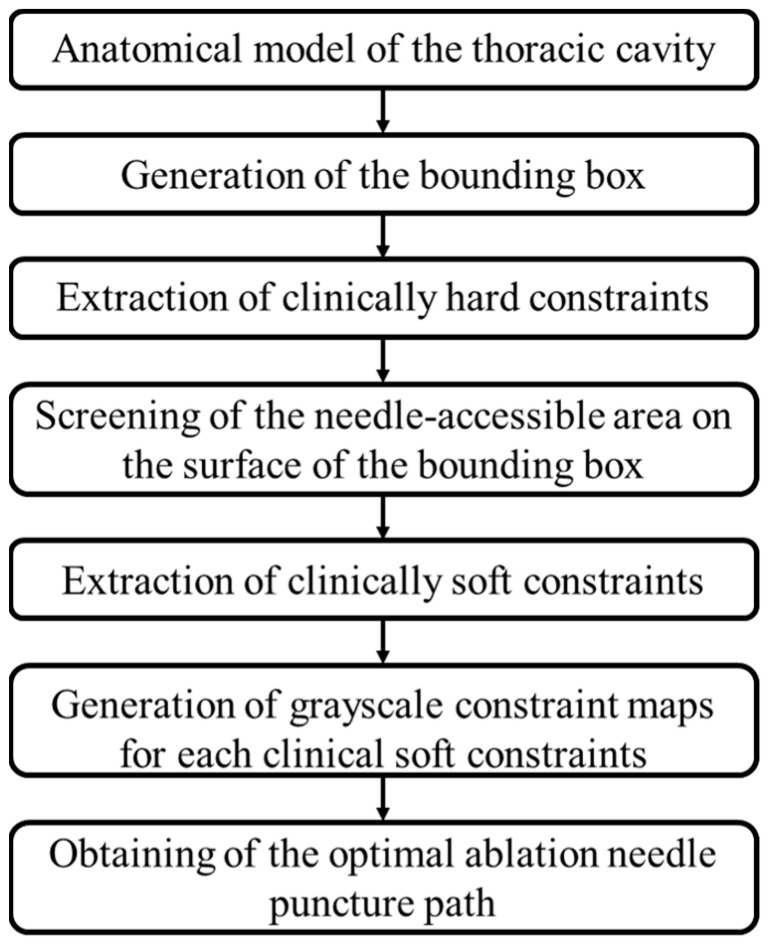
Flow chart of the proposed needle insertion trajectory planning method for lung tumor ablation.

**Figure 3:**
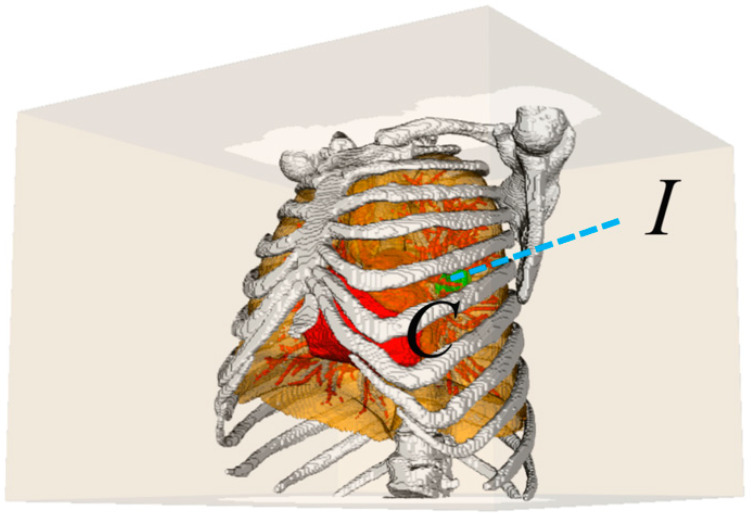
Schematic diagram of potential insertion trajectory. The cube indicates the patient’s external bounding box, whose internal structures are the anatomical scene of the patient’s thoracic cavity. Point *I* indicates a point on the bounding box (potential insertion point). Point *C* indicates the tumor centroid (target point). The blue dotted line corresponds to the potential insertion trajectory.

Planning of needle or application insertion path is critical to thermal tumor ablations (Figure 1). This study aims to propose an automatic needle insertion path-planning method for lung tumor ablation. The flow chart of the proposed method is shown in Figure 2. First, a bounding box was generated based on the anatomical structure model of the chest, and the line connecting each point on the bounding box to the center of the lesion served as a potential path (Figure 3). Subsequently, the hard constraints were extracted and the feasible needle area on the surface of the bounding box was extracted by the improved cube mapping method. Then, the soft constraints were extracted and the grayscale constraint maps of each soft constraint were generated by the improved cube mapping method, for which the degree of conformity of each potential path to each soft constraint was saved in the form of voxel values on the surface of the bounding box. Finally, based on the Pareto optimization algorithm, the feasible needle path with various soft constraint evaluation characteristics was optimized, and the optimal needle path was obtained.

### 2.1. Clinical Constraints

With the development of medical image processing and visualization techniques, researchers have quantified the clinical needs and goals of ablation therapy into several clinical constraints, which are planning criteria for computer-aided path-planning algorithms. Based on the literature related to ablation planning [16] and lung puncture biopsy [22], clinical constraints for thermal ablation of the lung tumor are divided into two categories: hard constraints and soft constraints. The hard constraints are the conditions that must be met for path planning; otherwise, it will directly lead to the failure of path planning. With the hard constraints, the region where the needle can be inserted can be screened out on the patient’s chest surface. The soft constraints are preferred conditions that need to be met as much as possible in the path-planning process. It is possible to optimize the optimal puncture path in the area where needle penetration is available.

1. Hard constraints

H1. The planned path should avoid vital organs and tissues that should not be touched in the thoracic cavity, including bones, trachea, vessels, interlobular fissures, and mediastinum.

H2. The percutaneous puncture length of the planned path should be strictly less than the ablation needle length.

H3. The angle between the planned path and the surface of the lung parenchyma should be at least greater than the usual clinical threshold (20°) to avoid needle slippage.

H4. The planned path should have enough depth of insertion into the lung (>5 mm) for hemostasis and ablation needle fixation.

2. Soft constraints

S1. The planned path should be as far as possible from the vital organs and tissues in the chest cavity that should not be touched.

S2. The percutaneous puncture length of the planned path should be as short as possible.

S3. The angle of the planned path to the lung parenchyma should be as large as possible.

### 2.2. Improved Cube Mapping Method

In this study, a cube mapping algorithm based on perspective projection was used to establish the link between the puncture path and the clinical constraints. The cube mapping method consists of six perspective projections, each of which is equivalent to a perspective camera located at the center of the projection taking a picture and recording the results. The perspective projections take the positive/negative directions of the three orthogonal coordinate axes of the Cartesian coordinate system as the focal directions, and the field of view is fixed, rendering the scene information in all directions along each line of sight originating from the center of the projection.

**Figure 4:**
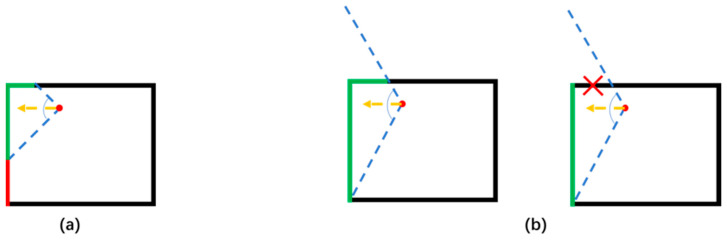
Cause of coordinate error and its correction strategy between traditional cube mapping and bounding box surface of the scene. The yellow arrow indicates the focus direction. The blue dotted line indicates the projection angle. The red dot indicates the projection center and the cube frame indicates the bounding box of the environment. (**a**) Cause of coordinate error. (**b**) The error correction strategy proposed in this paper.

The traditional cube mapping algorithm has two prerequisites: (1) The camera needs to be fixed at the center of the cube environment; (2) the conical viewing angle of the camera is fixed at 90°. However, in the actual ablation procedure, as shown in Figure 4a, the tumor is not located in the center of the patient’s thoracic cavity, and fixing the conical view angle of the camera causes the projection plane to be misaligned. To this end, a correction strategy is designed in this study, as shown in Figure 4b. Firstly, the focal length of the perspective camera is kept parallel to the Cartesian coordinate axis, and then the viewing angle is increased adaptively to ensure that the scene information in the corresponding plane of the bounding box can be completely captured by one perspective projection. Finally, the redundant information in the perspective projection result that is not the current surface is deleted, so that the spatial coordinate system of the cube mapping result can be realized as a one-to-one correspondence with the spatial coordinates of the bounding box.

### 2.3. Filtering of Feasible Needle Area Based on Hard Constraints

Based on hard constraints, a method for delineating the feasible needle area is proposed. The inputs to the algorithm include the skin of the patient’s chest cavity, lungs, bones, blood vessels, trachea, heart, and lung tumors, and the output of the algorithm is the feasible needle area delineated on the surface of the bounding box of the patient’s CT image.

#### 2.3.1. The Planned Path Should Avoid Vital Organs and Tissues (H1)

**Figure 5:**
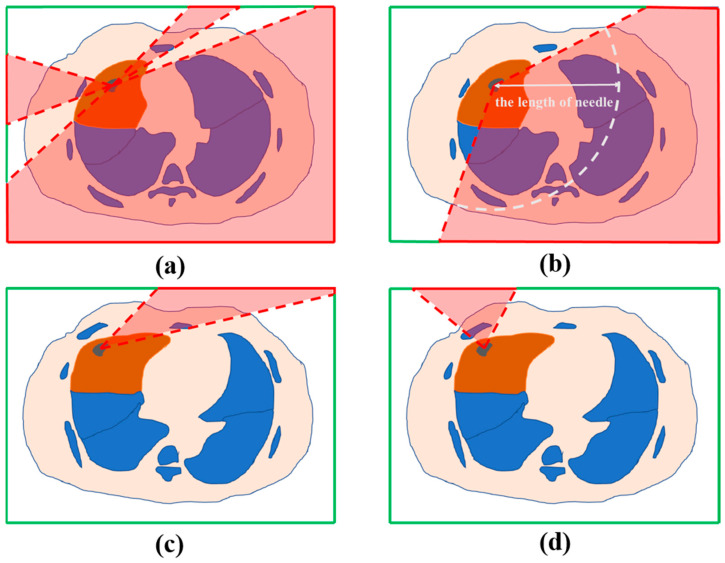
Schematic diagram of filtering of feasible needle area based on hard constraints H1 (**a**), H2 (**b**), H3 (**c**), and H4 (**d**). The lung tumor, the lung lobe containing the tumor, and bones, along with other structures that should not be contacted, are represented by areas in gray, orange, and blue, respectively. The transparent red coverage area indicates the non-feasible needle insertion area, while areas without the red overlay represent the feasible needle insertion area.

Considering the comprehensive accuracy and efficiency, the marching cubes algorithm was used to extract the equivalent surfaces for the 3D surface reconstruction of the organs and tissues at risk that needed to be avoided, and the anatomical environment of the patient’s thoracic cavity was obtained. The vital structure was then mapped to the bounding box by perspective projection using the improved cube mapping algorithm. During the projection process, the projection camera was placed at the center of the tumor, and perspective projections were made toward each of the six orthogonal surfaces of the patient’s CT image, which were taken up by the corresponding surface of the bounding box. Finally, the results of cube mapping were classified into two categories: The area covered by the vital structures was regarded as the non-feasible needle area, and the remaining areas were regarded as the feasible needle area. For ease of understanding, the risk structures are exemplified only by the bones and other lobes of the lungs, which are shown in a 2D schematic in Figure 5a.

#### 2.3.2. The Percutaneous Puncture Length of the Planned Path Should Be Strictly Less Than the Ablation Needle Length (H2)

As shown in Figure 5b, after calculating the distance of each voxel within the chest skin voxel set from the tumor center, the area of voxels that satisfied H2 was removed, and the remaining set of body surface voxels constituted the risk area that did not satisfy H2, which was cube-mapped to mark the non-feasible needle area on the surface of the bounding box.

#### 2.3.3. The Angle between the Planned Path and the Surface of the Lung Parenchyma Should Be Greater Than the Usual Clinical Threshold (H3)

As shown in Figure 5c, the realization of H3 also requires traversing the voxel collection of the patient’s chest skin, calculating the angle *α* between the direction vector of the connection between each voxel and the tumor center and the corresponding lung parenchymal surface, and removing the regions of voxels with an angle of greater than 20°. The remaining set of voxels constituted a region that did not satisfy H3, which was cube-mapped to mark the non-feasible needle area on the surface of the bounding box.

Angle *α* could not be calculated directly, and the normal vector on the surface of the lung parenchyma needed to be utilized. First, the reconstruction result of the lung parenchyma was subdivided into polygon meshes. Subsequently, the ID of the surface unit where the collision occurred was locked, and angle *β* between the surface normal vector of the unit and the potential puncture path was calculated. Finally, the included angle *α* was obtained through the supplementary angle relationship.

#### 2.3.4. The Planned Path Should Have Enough Depth of Insertion into the Lung (H4)

As shown in Figure 5d, the red area on the border of the bounding box corresponded to the area where the insertion depth of the lung was shallow, that is, the non-feasible area that did not satisfy H4. In our implementation, the expected ablation area was expanded by 5 mm, and the expanded part beyond the lung parenchyma was marked as the vital structure, which was cube-mapped to mark the non-feasible needle area on the surface of the bounding box.

#### 2.3.5. Integration of Hard Constraints H1–H4

**Figure 6:**
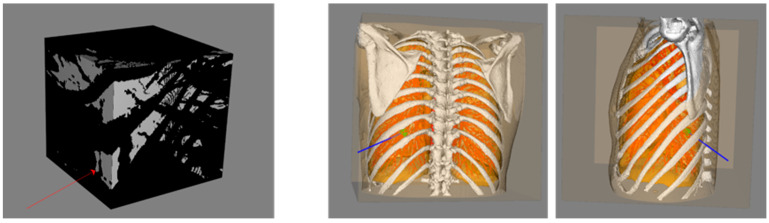
Schematic diagram of the bounding box integrating H1–H4 and its multi-angle verification. The blue line indicates the connection from the needle insertion point indicated by the red arrow to the center of the tumor.

An OR operation on the bounding box for the above four non-feasible areas was performed, and then the final feasible needle area could be obtained on the surface of the bounding box of the patient’s CT image (the gray value of the area blocked by the risk area was 0, and the gray value of the unblocked area was 1). As shown in Figure 6, on the final bounding box, an arbitrary point was selected in the area with a gray value of 1 as the needle insertion point to connect to the center of the tumor. The corresponding path was then plotted in a 3D anatomical structure for verification, and the path passed through the rib gap without violating any of the hard constraints.

### 2.4. Path Optimization Based on Soft Constraints

The line connecting each coordinate within the feasible needle area and the centroid of the tumor can be regarded as a potential puncture path, but one or several optimal puncture trajectories need to be selected among the potential puncture paths. To this end, this study established corresponding scoring standards for each soft constraint involved. The Pareto optimization and weighted summation were used to solve multi-objective optimization problems, and the optimized paths were evaluated. The potential path with the highest score was regarded as the final planned path.

#### 2.4.1. The Planned Path Should Be as Far as Possible from the Vital Organs and Tissues (S1)

**Figure 7:**
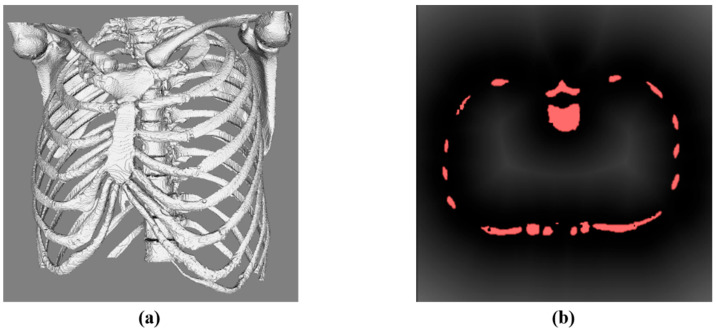
Schematic diagram of distance transformation results of vital structures (bone). (**a**) 3D model of bone. (**b**) The corresponding result of DTF (an axial slice).

To satisfy the soft constraint S1, it is necessary to obtain the actual distance between the potential planning path and the vital structure. First, the 3D distance transformation (DTF) algorithm [23] was used to calculate the actual distance between each coordinate in the space and the vital structure, and the intensity of the gray value was used to indicate the distance. Figure 7b shows an axial slice of the DTF result using only the bone for demonstration. For ease of understanding, the actual bone (marked in red) is displayed on the DTF result. The closer the voxel is to the bone, the lower the corresponding gray value. As the spatial voxel moves away from the bone, the corresponding gray value is higher.

Then the minimum density projection (MinIP) volume was drawn on the result of DTF to obtain the minimum distance between each potential path and the vital structure. This process is equivalent to traversing the distance between each point on the potential puncture path and the vital structure and returning the minimum value among these distances as the actual distance between the potential path and the vital structure. Finally, the improved cube mapping method proposed in this study was used to project the MinIP volume rendering results on the surface of the bounding box in perspective. The gray value of each coordinate is the actual distance between the potential puncture path and the vital structure using that coordinate as the puncture starting point.

Then, the gray value of the bounding box was normalized according to Equation (1). This kind of image, which uses the intensity of the gray value to reflect the degree to which the corresponding planned path satisfies the constraint conditions, is called a grayscale constraint map in this study.

(1) $$R_{i}=\frac{d_{i}-d_{min}}{d_{max}-d_{min}}$$

where *d_i_* is the gray value of the distance between the current path *i* and the vital structures in the gray constraint map corresponding to S1. *d_min_* and *d_max_* are the minimum and maximum gray values in the gray constraint map of S1.

#### 2.4.2. The Percutaneous Puncture Length of the Planned Path Should Be as Short as Possible (S2)

**Figure 8:**
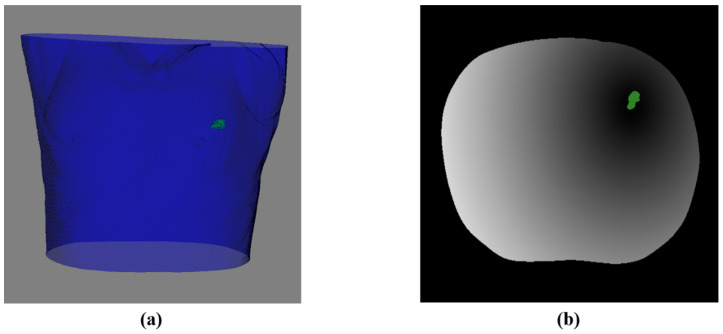
Schematic diagram of DTF results of a lung tumor. (**a**) 3D model of the skin and tumor. (**b**) The corresponding result of DTF after processing (an axial slice).

To satisfy the soft constraint S2, it was necessary to obtain the actual distance from the skin needle insertion point corresponding to the potential planning path to the tumor center. First, DTF was performed based on the lung tumor centroid to obtain the distance transformation result (Figure 8), and the part of the result that exceeded the chest cavity surface was set to the lowest gray value.

The length of the puncture path can be considered as the maximum distance between each point traversed by the puncture path and the tumor center. Therefore, maximum intensity projection (MIP) volume rendering was used. Finally, the improved cube mapping was performed on the volume rendering result to obtain the grayscale constraint map corresponding to S2. The bounding box was normalized with Equation (2), and *L_i_* was obtained as the potential path’s satisfaction score for S2.

(2) $$L_{i}=1-\frac{l_{i}-l_{min}}{l_{max}-l_{min}}$$

where *l_i_* is the gray value of the percutaneous puncture length of the current path *i* in the gray constraint map corresponding to S2. *l_min_* and *l_max_* are the minimum and maximum gray values in the gray constraint map of S2.

#### 2.4.3. The Angle of the Planned Path to the Lung Parenchyma Should Be as Large as Possible (S3)

The soft constraint S3 can be directly implemented on the surface of the bounding box, and the line connecting each point on the surface of the bounding box and the tumor centroid is regarded as a potential planning path. The angle between the path and the surface of the lung parenchyma was calculated and stored in the corresponding coordinates as a gray value. Finally, the grayscale constraint map corresponding to S3 was obtained. The bounding box was normalized with Equation (3), and *A_i_* was obtained as the potential path’s satisfaction score for S3.

(3) $$A_{i}=1-\frac{\alpha_{i}-\alpha_{min}}{\alpha_{max}-\alpha_{min}}$$

where $\alpha_{i}$ is the gray value of the angle between the current path *i* and the surface of the lung parenchyma in the gray constraint map corresponding to S3. $\alpha_{min}$ and $\alpha_{max}$ are the minimum and maximum gray values in the gray constraint map of S3.

#### 2.4.4. Optimal Path Calculation Based on Pareto Optimization

For the three gray-scale constraint maps obtained, the gray-scale value of each coordinate point (corresponding to each path) represents the degree of compliance of the path under a certain soft constraint condition. Then a comprehensive evaluation system needed to be established to select the optimal path. This is a multi-objective optimization task [24]. This study adopted the method of Pareto optimization combined with weighted summation. First, the Pareto front set was selected based on Pareto optimization, which represents the solution set that cannot be further optimized. Then, through the weighted integration of three soft constraints, the path with the highest score in the Pareto front solution set was taken as the final planned path.

**Figure 9:**
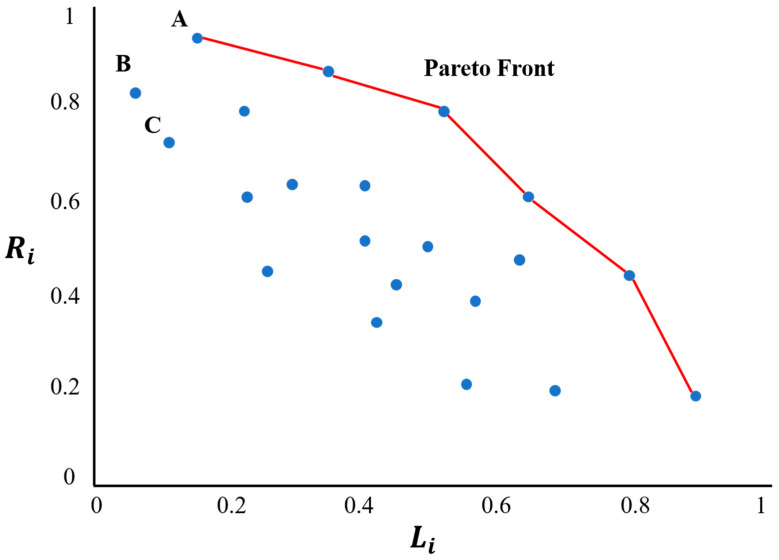
Schematic diagram of the Pareto coordinate system established with soft constraints S1 and S2. *R_i_* from S1 is taken as the vertical axis and *L_i_* from S2 is taken as the horizontal axis.

Pareto optimization [25] is a method widely used in multi-objective optimization. It describes a state of optimal allocation of resources. Taking soft constraints S1 and S2 as examples, as illustrated in Figure 9, the coordinate system is constructed with two soft constraints: “the distance from the path to the vital structures:$\;R_{i}$” and “the length of the path:$\;L_{i}$”. The blue points in the coordinate system represent the set of needle entry points within the feasible needle area. The set of points on the red connecting line is regarded as the Pareto front under this coordinate system. The specific steps to generate the Pareto front set under this coordinate system are as follows:

1. Integration of soft constraints: Elements from the sets corresponding to soft constraints S1 (each evaluated by $R_{i}$) and S2 (each evaluated by $L_{i}$) are merged on a per-element basis. This unified set serves as the input for the subsequent algorithmic processing.
2. Initialization: The Pareto front set, designated as the resultant set, is initially established as an empty entity.
3. Sorting: The elements within the set are organized in a descending sequence, prioritized first by $R_{i}$ and subsequently by $L_{i}$.
4. Selection and pruning: The element exhibiting the maximal $R_{i}$ value from the pool of unprocessed elements (e.g., Path A) is selected and incorporated into the Pareto front set. Concurrently, elements within the input set possessing $L_{i}$ values inferior to that of the selected element (e.g., Path B and C) are eliminated.
5. Iterative process: The selection and pruning step is reiterated until there is a convergence between the elements of the input set and those in the result set.
6. Output: The Pareto front set is then outputted.

For these coordinate points, there is no coordinate point that is better in both indicators. Therefore, it is regarded as the optimal needle entry point set under these two soft constraints. Furthermore, for all soft constraints, a pairwise relationship is established, and then the intersection of the obtained Pareto front solution sets is obtained to attain the Pareto optimal point set in the global scope.

The Pareto optimal solution is an acceptable solution set for the problem. Generally, there are multiple Pareto optimal solutions, and there is no distinction between the advantages and disadvantages of each solution. Therefore, we used the weighted product form to integrate the sub-goal constraints according to Equation (4), and the points in the Pareto optimal solution set were brought into it to obtain the comprehensive score of each optimal solution. The highest *G_i_* was taken as the final planned path:

(4) $$G_{i}=\lambda_{1}*R_{i}+\lambda_{2}*L_{i}+\lambda_{3}*A_{i}$$

where $\lambda_{1}–\lambda_{3}$ are the weight factors of clinical soft constraints S1–S3, respectively. The weight factors were initialized as $\lambda_{1}=\lambda_{2}=\lambda_{3}=1/3$ and could be modified when necessary.

## 3. Results

**Figure 10:**
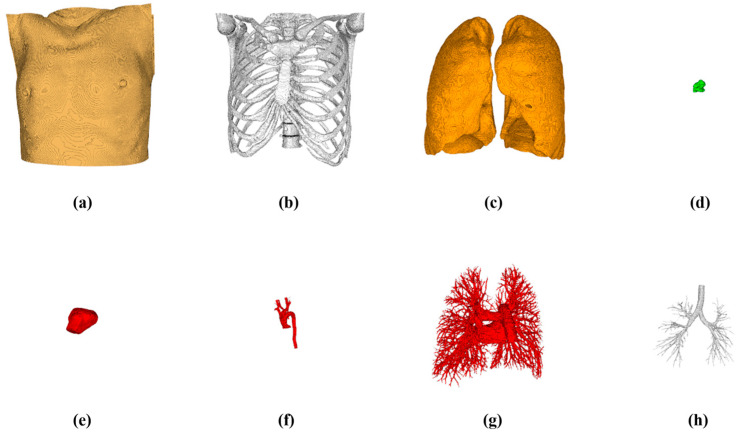
Visualization of input data for each part. (**a**) Skin. (**b**) Bones. (**c**) Lung parenchyma. (**d**) Lung tumor. (**e**) Heart. (**f**)Aortic arch. (**g**) Lung vessels. (**h**) Bronchi.

**Figure 11:**
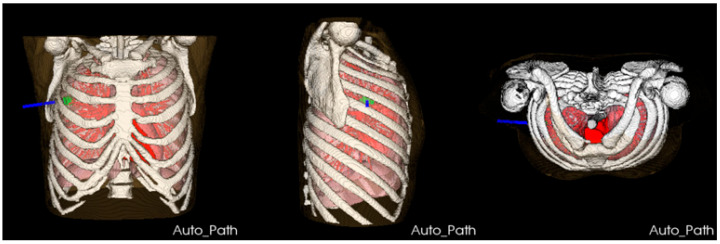
A case of path-planning results. The green area indicates a lung tumor. The blue line indicates the automatically planned ablation needle puncture path.

The clinical thoracic CT data of 15 patients with lung tumors, suitable for single-needle thermal ablation (tumor diameter < 3 cm), were selected from the Beijing Chest Hospital as data sets. Among these patients, 3 of them had multiple tumors, with a total of 18 lung tumors of varying sizes, ranging from 482.79 mm^3^ to 9313.81 mm^3^. These data sets were used to retrospectively verify the proposed ablation needle puncture path-planning method. The number of pixel points of a single-layer image is 512 × 512, and the layer thickness is 1.25 mm. The number of slices of CT images per case ranged from 221 to 463. As illustrated in Figure 10, experts manually segmented a variety of thoracic structures from the CT data, including the skin, bone, lung parenchyma, lung tumor, heart, pulmonary trunk, vessels, and bronchi, to construct a comprehensive thoracic anatomy scene that serves as the input for the algorithm. Then the automatic ablation needle puncture path-planning algorithm was performed on the 18 lung tumors. The algorithm was implemented with the C++ language under Visual Studio 2017, and the corresponding visualization and image processing functions were implemented based on the Visualization Toolkit (VTK) and Insight Segmentation and Registration Toolkit (ITK). In the desktop computer HP Z4 (Intel Xeon W-2223, 3.60 GHz, 32 GB RAM, Windows 10, 64-bit), the average puncture planning time of the algorithm for 18 tumors was 35 s. A case was randomly selected to execute the designed algorithm. A three-view view of the result is shown in Figure 11.

Since there is no absolute gold standard for ablation needle puncture path planning, we referred to other related studies [20,22] and invited two experienced clinicians to evaluate the performance of the proposed path-planning method.

### 3.1. Quantitative Evaluation of Planning Results

The proposed method was used to automatically plan needle paths, and the top five puncture paths in each tumor were retained. The paths that met all hard constraints were considered qualified paths, and the number of qualified paths and the pass rate in each case were calculated. After discussion with clinicians, we quantified the soft constraints based on the hard constraints and formulated excellent standards as follows: “the distance between the path and vital structures is greater than 10 mm”, “the path length is less than 100 mm”, and “the needle insertion angle is greater than 70°”. The number and excellence rate of the puncture paths that meet the above-mentioned excellence standards 1, 2, and 3 in each case’s data were calculated, respectively. The results are shown in Table 1.

The results showed that the pass rate of the 90 planned puncture paths for the 18 cases was 100%, indicating that none of the planned paths violated any hard constraints. The overall excellent rate was 61.11%, the excellent rate of the sixth case was 0%, and the needle entry angle of the top five paths did not reach 70° (67°, 60°, 59°, 62°, 54°). After reviewing the data, it was found that the tumor location in this case was complex and close to the bone. It was impossible to find a path with a needle insertion angle greater than 70° within the feasible needle insertion area. Although the planned path did not meet the excellent standard, it was still acceptable. Except for the sixth exception, there was at least one planning path in other data that could meet the above excellent conditions, which demonstrated the effectiveness of the proposed path-planning method.

### 3.2. Qualitative Assessment of Planning Results

Two clinicians jointly evaluated the rationality of the automatic planned paths on the 3D model through their extensive clinical operation experience to count the number of reasonable paths and unreasonable paths. Clinicians were then invited to manually plan a puncture path based on the original CT image and add it to the set of automatically planned paths. Finally, the two clinicians independently ranked a total of six paths for each case, and one clinician’s evaluation was blind to the other clinician. The specific results are shown in Table 2.

The results showed that 82.22% of the paths were considered reasonable. Clinician A believed that the automatic planning results of nine cases were better than those of manual planning. Clinician B believed that the automatic planning results of seven cases were better than those of manual planning. Due to the different personal operating habits of clinicians, the chosen path in clinical practice may be different. However, both clinicians agreed that automatic planning was far more efficient than manual planning (only taking about 35 s) and could generate a path ranking list for clinicians’ reference, which could be used as a computer-aided means. The automatic path-planning method not only reduces the preoperative planning workload of clinicians, but also has certain practical value for clinicians (especially for those with less experience).

## 4. Discussion

We propose a CT-guided preoperative path-planning method for thermal ablation of lung tumors, which obtains the feasible needle area based on hard constraints and optimally plans the puncture path in the feasible needle area based on soft constraints. Our method can provide clinicians with an accurate and reasonable puncture path reference for preoperative diagnosis. Since there are few studies related to lung tumor ablation path planning, we summarized the related work on liver tumor ablation path planning and lung biopsy path planning, and compared it with our work, the results of which are summarized in Table 3.

Although there is no gold standard for evaluating ablation needle puncture path-planning methods, the effectiveness of the proposed method can still be evaluated based on the implementation of the ablation needle puncture path-planning method. Our method has the following advantages.

1. Some related studies [14,17] involve drawing isosurfaces of the skin and subdividing these into polygonal meshes, using lines from mesh vertices to the puncture target to form potential paths. The number and distribution of these paths, typically ranging from 2000 to 6000, depend on the mesh quality. Another study [23] generated potential paths using discrete deflection angles. When the degree of deflection is fixed at 1°, 360^2^ potential paths can be produced. However, both this method and direct skin surface sampling [22] suffer from uneven sampling. Our method uses the bounding box of the patient’s CT image to generate potential paths. The number and accuracy of potential puncture paths are only positively related to the spatial sampling of the patient’s CT image. Compared with other methods, our method generates a larger number of potential paths and is more evenly distributed. At the same time, the time efficiency is not significantly reduced. Therefore, from the perspective of generating potential paths, our method has certain advantages.
2. When extracting hard/soft constraints, related studies [14,17] on the rendering of vital structures using polygon mesh surfaces involve applying collision detection through an exhaustive method (performed at least 2000–6000 times) to evaluate how well potential paths adhere to constraints. By contrast, our method designs an improved cube map method based on perspective projection. For each soft constraint, only six projections are needed without traversing the entire skin. In the specific implementation, multiple grayscale constraint maps are generated in parallel, which greatly improves planning efficiency.
3. In terms of dealing with optimization problems with multiple clinical constraints, the simplex algorithm is sensitive to the influence of the initialization starting point. The single-weighted summation method necessitates the pre-setting of weights artificially, potentially leading to less objective outcomes. The single Pareto optimal method yields multiple solution sets rather than a single optimal solution, necessitating clinicians to engage in interactive operations across various Pareto fronts to identify locally optimal solutions. Our method uses a combination of Pareto and weighted summation when optimizing the paths, which achieves relatively objective and rational automatic planning of the ablation needle path.
4. Our method has good scalability and can add constraints according to actual needs. During specific implementation, it only needs to generate a new grayscale constraint map and add it to the optimization model.

However, there are still some limitations.

1. Our method belongs to single-needle path planning and is suitable for lung tumors with a diameter of less than 3 cm. Large tumors usually require multiple single-needle or multiple-needle single-shot ablation, which requires comprehensive planning combined with a simulation model of the coagulation zone that changes dynamically over time.
2. Our method belongs to “linear” path planning and does not consider the interactive deformation (tissue deformation and ablation needle deformation) produced when the ablation needle is inserted into the patient’s body. This is not only because these deformation factors are difficult to model, but also because the impact of these deformation factors on path planning is difficult to reasonably integrate into “straight-line” path planning. This is also a challenge for related work in this field.

In the future, the path-planning method will be integrated with another research branch of our laboratory, determining the treatment parameters (e.g., ablation power and duration) through temperature field simulation to provide a more comprehensive preoperative plan. We will use additional clinical data to validate and improve our method. In addition, the optimal path provided by this method can also be used as a key part of the preoperative software planning of surgical robots, coordinating spatial registration, navigation, and other links to jointly improve the accuracy of surgical robots.

## 5. Conclusions

We propose a puncture path-planning method for thermal ablation of lung tumors. Firstly, the feasible puncture area was determined based on clinical hard constraints. On the basis of the feasible puncture area, grayscale constraint maps corresponding to the clinical soft constraints were constructed. Finally, the puncture path was planned by combining Pareto optimization and a weighted summation algorithm. We conducted experimental verifications on 18 lung tumors of varying sizes (482.79 mm^3^ to 9313.81 mm^3^) and invited experienced clinicians to evaluate the planning path. The findings indicated that over 82% (74 of 90) of the paths were deemed reasonable. Overall, the results of the automatic path planning were comparable to those of manual planning by experienced clinicians. However, our method significantly reduces the time and workload for clinicians, taking only 35 s, and providing preoperative path plans more quickly. This is particularly beneficial for inexperienced clinicians. In the future, we plan to consider the impact of respiration on internal organ displacement during actual ablation surgeries to further enhance the accuracy of our path planning in actual operations and use more data for verification. Additionally, this method will be integrated with another research branch of our laboratory, determining the treatment parameters (e.g., ablation power and duration) through temperature field simulation to provide a more comprehensive preoperative plan.

## Figures and Tables

**Table 1:** Quantitative evaluation of path-planning results.

Tumor ID	Qualified Quantity	Pass Rate	Excellent Quantity	Excellent Rate
1	5	100%	4	80%
2	5	100%	1	20%
3	5	100%	2	40%
4	5	100%	5	100%
5	5	100%	2	40%
6	5	100%	0	0%
7	5	100%	2	40%
8	5	100%	3	60%
9	5	100%	3	60%
10	5	100%	4	80%
11	5	100%	1	20%
12	5	100%	2	40%
13	5	100%	5	100%
14	5	100%	4	80%
15	5	100%	4	80%
16	5	100%	1	20%
17	5	100%	3	60%
18	5	100%	4	80%

**Table 2:** The comparison of the manual path and automatically planned paths using evaluations by two experienced clinicians blind to each other.

Tumor ID	Reasonable Quantity	Unreasonable Quantity	Ranking of the Manual Path	
			Clinician A	Clinician B
1	5	0	2/6	1/6
2	4	1	2/6	1/6
3	3	2	1/6	1/6
4	5	0	2/6	1/6
5	4	1	3/6	2/6
6	3	2	1/6	1/6
7	4	1	2/6	2/6
8	5	0	1/6	1/6
9	5	0	2/6	3/6
10	3	2	1/6	2/6
11	4	1	1/6	1/6
12	5	0	2/6	1/6
13	4	1	2/6	1/6
14	5	0	1/6	2/6
15	4	1	2/6	1/6
16	4	1	1/6	2/6
17	3	2	1/6	1/6
18	4	1	1/6	3/6

**Table 3:** Comparison of the needle insertion trajectory planning method proposed in this paper with related work.

Authors	Application	Potential Paths Generation Methods	Optimization Methods
Villard et al. [14]	Liver tumor ablation	Polygons based on skin fitting	Simplex algorithm
Baegert et al. [17]	Liver tumor ablation	Polygons based on skin fitting	Semi-exhaustive algorithm
Schumann et al. [23]	Liver tumor ablation	Sampling on skin using deflection angles	Weighted summation
Bao et al. [22]	Lung biopsy surgery	Raw skin voxels	Pareto optimization
Ours	Lung tumor ablation	Bounding box of the patient’s CT image	Pareto optimization and weighted summation

## Data Availability

The raw data supporting the conclusions of this article may be provided upon reasonable requests for scientific research purposes.
